# Computational analysis of cortical neuronal excitotoxicity in a large animal model of neonatal brain injury

**DOI:** 10.1186/s11689-022-09431-3

**Published:** 2022-03-29

**Authors:** Panagiotis Kratimenos, Abhya Vij, Robinson Vidva, Ioannis Koutroulis, Maria Delivoria-Papadopoulos, Vittorio Gallo, Aaron Sathyanesan

**Affiliations:** 1 Center for Neuroscience Research, Children’s National Research Institute, Children’s National Hospital, 111 Michigan Avenue, Washington, DC, 20010 USA; 2 Department of Pediatrics, Division of Neonatology, Children’s National Hospital, Washington DC, USA; 3 George Washington University School of Medicine and Health Sciences, Washington DC, USA; 4 Digirobi Solutions, Bengaluru, Karnataka India; 5 Department of Pediatrics, Division of Emergency Medicine, Children’s National Hospital, Washington, DC, USA; 6 Center for Genetic Medicine Research, Children’s National Research Institute and Department of Genomics and Precision Medicine, George Washington University School of Medicine and Health Sciences, Washington, DC, USA; 7 Department of Pediatrics, Drexel University College of Medicine, Philadelphia, PA USA

**Keywords:** Neonatal brain injury, Excitotoxicity, Src kinase, Calcium/calmodulin, Nuclear calcium, Computational modeling, SimBiology

## Abstract

**Background:**

Neonatal hypoxic brain injury is a major cause of intellectual and developmental disability. Hypoxia causes neuronal dysfunction and death in the developing cerebral cortex due to excitotoxic Ca^2+^-influx. In the translational piglet model of hypoxic encephalopathy, we have previously shown that hypoxia overactivates Ca^2+^/Calmodulin (CaM) signaling via Sarcoma (Src) kinase in cortical neurons, resulting in overexpression of proapoptotic genes. However, identifying the exact relationship between alterations in neuronal Ca^2+^-influx, molecular determinants of cell death, and the degree of hypoxia in a dynamic system represents a significant challenge.

**Methods:**

We used experimental and computational methods to identify molecular events critical to the onset of excitotoxicity-induced apoptosis in the cerebral cortex of newborn piglets. We used 2–3-day-old piglets (normoxic [Nx], hypoxic [Hx], and hypoxic + Src-inhibitor-treatment [Hx+PP2] groups) for biochemical analysis of ATP production, Ca^2+^-influx, and Ca^2+^/CaM-dependent protein kinase kinase 2 (CaMKK2) expression. We then used SimBiology to build a computational model of the Ca^2+^/CaM-Src-kinase signaling cascade, simulating Nx, Hx, and Hx+PP2 conditions. To evaluate our model, we used Sobol variance decomposition, multiparametric global sensitivity analysis, and parameter scanning.

**Results:**

Our model captures important molecular trends caused by hypoxia in the piglet brain. Incorporating the action of Src kinase inhibitor PP2 further validated our model and enabled predictive analysis of the effect of hypoxia on CaMKK2. We determined the impact of a feedback loop related to Src phosphorylation of NMDA receptors and activation kinetics of CaMKII. We also identified distinct modes of signaling wherein Ca^2+^ level alterations following Src kinase inhibition may not be a linear predictor of changes in Bax expression. Importantly, our model indicates that while pharmacological pre-treatment significantly reduces the onset of abnormal Ca^2+^-influx, there exists a window of intervention after hypoxia during which targeted modulation of Src-NMDAR interaction kinetics in combination with PP2 administration can reduce Ca^2+^-influx and Bax expression to similar levels as pre-treatment.

**Conclusions:**

Our model identifies new dynamics of critical components in the Ca^2+^/CaM-Src signaling pathway leading to neuronal injury and provides a feasible framework for drug efficacy studies in translational models of neonatal brain injury for the prevention of intellectual and developmental disabilities.

**Supplementary Information:**

The online version contains supplementary material available at 10.1186/s11689-022-09431-3.

## Background

Cerebral hypoxia in neonates is a potent driver of excitotoxic neuronal injury that often leads to cell death [1]. Neuronal death during this early developmental period causes long-term alterations in neural circuits, resulting in poor intellectual and developmental outcomes later in life [2]. However, only few targeted therapeutic strategies currently exist to prevent or ameliorate excitotoxicity and associated neuronal injury to the neonatal cerebral cortex, due to the lack of mechanistically detailed studies in translationally relevant models of neonatal brain injury [3].

Neuronal excitotoxicity is strongly linked to abnormal Ca^2+^ influx due to uncontrolled glutamatergic drive [4]. The canonical understanding of neuronal excitotoxicity suggests that a sustained disruption of Ca^2+^ homeostasis, due to overactivity of ionotropic N-methyl d-aspartate (NMDA) and α-amino-3-hydroxy-5-methyl-4-isoxazolepropionic acid (AMPA)/kainate receptors, eventually leads to the onset of mitochondrial-dependent or independent cell death pathways [2]. In this framework, cell death is a consequence of an overload on neuronal Ca^2+^ buffering systems [5], such as the Ca^2+^/CaM signaling cascade [6, 7]. Evidence for this framework draws from hypoxic injury in the adult brain. However, injury during the neonatal period is complex, due to the effects of multiple signaling cascades related to both physiological development and/or pathology being differentially affected [8]. As a result, the specific mechanisms that link overloaded Ca^2+^ buffering to the onset of cell-death-related gene expression have not yet been precisely and systematically defined in the context of neonatal injury of the cerebral cortex.

Past efforts to rescue brain-hypoxia-induced excitotoxic mechanisms have been focused on preventing abnormal Ca^2+^ influx by targeting the ionotropic mode of NMDAR activity [9, 10]. These efforts have largely been unsuccessful [9, 11]. While targeting the ionotropic mode of NMDAR activity has not succeeded in clinical trials, emerging evidence points to another avenue for therapeutic intervention in hypoxic neuronal injury: non-ionotropic or quasi-metabotropic modes of NMDAR function [12]. In particular, the interaction between sarcoma (Src) kinase and NMDARs shows significant potential as a therapeutic target in ameliorating the effect of excitotoxic mechanisms induced by hypoxic insult to neurons [13]. Since Src kinase and its family members (Src family kinases; SFKs) also interact with ionotropic modes of NMDA function in non-pathological contexts [14], potential cross-talk could exist between Src and the two modes of NMDAR function.

In a translationally relevant piglet model of neonatal brain injury, we have established that hypoxia triggers Ca^2+^ influx into both the cytoplasm and nucleus [15]. Ca^2+^ influx leads to the formation of the activated Ca^2+/^CaM complex, which subsequently leads to the activation and translocation of Ca^2+^/CaM-dependent protein kinase IV (CaMKIV) to the nucleus, resulting in cyclic-AMP response element binding protein (CREB)-mediated transcription of apoptotic genes *Bax* and *Bcl2* [16, 17]. Studies from other mammalian models have shown that Ca^2+^ influx into the nucleus also directly modulates CREB-mediated gene regulation through CaMKIV [18]. We have also demonstrated that Src kinase is a critical component of Ca^2+^-dependent transcription of proapoptotic genes in the piglet model [19]. Pharmacological pretreatment of hypoxic piglets with PP2, a potent Src inhibitor, has a protective effect, keeping many of the Ca^2+^/CaM signaling components at normoxic levels and partially preventing the activation of CREB [19], further keeping the apoptotic machinery under control.

The failure to rescue the effect of excitotoxic mechanisms by solely focusing on NMDAR antagonism underscores the need for unified and integrated mechanistic approaches to determine effective therapeutics tailored to specific pathological and developmental contexts, such as neonatal hypoxic brain injury. While we have biochemically measured individual components of the Ca^2+^/CaM-Src pathway in normoxic, hypoxic, and treatment conditions, the relative importance of specific steps and reaction kinetics has not been explored. Further, we have not explored the potential role of other components in this and other convergent pathways which may be critical to overall Ca^2+^ homeostasis and cell-death-related processes. Therefore, in order to study a unified signaling pathway from receptor activation-mediated Ca^2+^ influx to proapoptotic protein expression, we built a computational model of the Ca^2+^/CaM-Src pathway. We calibrated our model using our previously published experimental data from the piglet model of neonatal brain injury and validated it using newly obtained experimental data on another critical yet unexplored component of the pathway: CaMKK2 in normoxic and hypoxic conditions. Simulations of our model indicate dissociable states between Ca^2+^ influx and Bax expression. Global sensitivity analysis reveals relative robustness to changes in concentration of CaMKIV, but significant sensitivity to glutamatergic hyperstimulation and activated CREB, suggesting an important role for convergent pathways in linking increased excitatory drive to apoptosis. Finally, our model provides a mechanistic basis for using interventions targeted to the feedback loop between Src and NMDAR.

## Methods

### Model construction

We constructed the computational model using the SimBiology Model Builder Interface (SimBiology version 6.1, MATLAB v. 2021a, Mathworks Inc., Natick, MA). A representation of our model is presented in Supplementary Fig. 1 (Supplementary material). Our model has four discrete compartments, each of which was considered well-mixed: extracellular space, cytoplasm with plasma membrane, nucleus with nuclear membrane, endoplasmic reticulum with endoplasmic reticulum membrane, and mitochondria. The model was built in a modular fashion and contains 7 integrated sub-models with their regulatory components:

1. Ca^2+^ sub-model, which incorporated movement of Ca^2+^ ions into and out of a particular compartment. The extracellular Ca^2+^ source (“Ca_ec”) was set as a constant. Ca^2+^ entry into the cytoplasm occurred via the NMDA receptor (NMDAR), voltage gated Ca^2+^ channel (VGCC), and the Ca^2+^-permeable AMPA receptor (CP-AMPAR). Ca^2+^ entry into the endoplasmic reticulum (ER) occurred through the SERCA pump, and movement of ER Ca^2+^ into the cytoplasm occurred via RyR and IP_3_/Ca^2+^ channels. The Na^+^/Ca^2+^ exchanger (NCX) modulated Na^+^ and Ca^2+^ movement between the nucleus and the ER
2. Src kinase sub-model, which included unphosphorylated, phosphorylated, and activated forms of Src, which also incorporated disinhibition of Src by protein tyrosine phosphatase nonreceptor type 1B (PTP-1B) [20]
3. Simplified oxygen-ATP sub-model
4. Simplified Ca^2+^/CaM cytoplasmic signaling cascade sub-model which included activated and non-activated forms of calcium/calmodulin (CaM), calcium/calmodulin-dependent protein kinase II (CaMKII), and calcium/calmodulin-dependent protein kinase kinase 2 (CaMKK2)
5. Ca^2+^/CaM nuclear signaling sub-model, which included nuclear-translocated activated and non-activated forms of Ca^2+^/CaM, CaMKII, CaMKK2, and calcium/calmodulin-dependent protein kinase IV (CaMKIV)
6. PKC-Raf-MEK-ERK cascade, which included unphosphorylated and phosphorylated forms of protein kinase C (PKC), and the mitogen-activated protein kinases Raf, MEK, and ERK
7. CREB-Creb binding protein (CBP) regulation of Bax transcription, which also included spliceosome processing [21].

The complete model was composed of 62 species, 134 reaction parameters, and 63 individual reactions. Reactions were modeled as either activation enzymatic reactions, where the enzyme *E* activates the molecule *A* at rate *k*_act_ to yield *A*_active_:

1 $$\mathrm{A}+\mathrm{E}\ \overset{\ {\mathrm{k}}_{\mathrm{act}}}{\to }{\mathrm{A}}_{\mathrm{act}\mathrm{ive}}+\mathrm{E}$$

or inactivation reactions, when *A*_active_ is inactivated at rate *k*_inact_:

2 $${\mathrm{A}}_{\mathrm{active}}\overset{\ {\mathrm{k}}_{\mathrm{inact}}\ }{\to}\mathrm{A}$$

or binding/unbinding reactions, when *A* binds to molecule *B* to yield *AB* at rate *k*_bind_, and unbinds at rate *k*_unbind_:

3 $$\mathrm{A}+\mathrm{B}\ \overset{\ {\mathrm{k}}_{\mathrm{bind}}}{\to}\mathrm{AB}$$

4 $$\mathrm{AB}\ \overset{\ {\mathrm{k}}_{\mathrm{unbind}}}{\to}\mathrm{A}+\mathrm{B}$$

or translocation/transport reactions, where *A* translocates from the cytoplasm into the nucleus at rate *k*_in_ and translocates out at rate *k*_out_:

5 $$\mathrm{A}\underset{{\mathrm{k}}_{\mathrm{out}}}{\overset{{\mathrm{k}}_{\mathrm{in}}}{\rightleftarrows }}{\mathrm{A}}_{\mathrm{nucleus}}$$

Ordinary differential equations (ODEs), starting quantities, fluxes for reactions, as well as supporting references are listed in the Supplementary material. The initial species concentrations were derived or estimated from other published models, our laboratory’s data, and from the conversion of label-free quantification signal intensity from localization data on PepTracker [22] to protein counts using the Perseus ‘Proteomics Microruler’ plug-in [23].

### Simulation of hypoxia, normoxia, and pharmacological intervention

We modeled conditions of normoxia (Nx) and hypoxia (Hx) by modifying the extracellular concentration of O_2_. Per Tuckerman et al. in normoxia, the intracellular O_2_ concentration in tissues is 10,000–40,000 ppm, in moderate hypoxia, [O_2_] = 1000–10,000 ppm, and in severe hypoxia [O_2_] = 0–1000 ppm [24]. Assuming a solvent density of 1 g/mol, these values correspond to 312.5–1250 mM, 31.25–312.5 mM, and 0–31.25 mM respectively. We used “Events” in the SimBiology model builder interface to set discrete transitions between Nx, Hx, and intervention with the Src-inhibitor PP2 (Hx+PP2). We set the hypoxic event trigger as time ≥ 120 s, with the event function lowering the extracellular O_2_ concentration to 3.25 μM. In the second event, we maintained Hx, but introduced PP2 at a concentration of 1 μM. For model input during Nx, we dosed extracellular glutamate at the amount defined by the parameter “stim,” for 12 pulses, with a 10 s inter-pulse interval. During Hx and Hx+PP2, we dosed extracellular glutamate at the amount defined by “hyperstim” delivering 48 pulses with a 5 s inter-pulse interval.

### Global sensitivity analysis (GSA) and multiparametric global sensitivity analysis (MPGSA)

We used the Global Sensitivity Analysis app [25] to perform GSA on our model and compute Sobol indices [26, 27]. We set the number of samples to 1000, and the output time to match the duration of our simulation: 0–360 s at a resolution of 1 s. The “interp1q” Interpolation method was used. Following computation of Sobol indices, the same simulations were reused for MPGSA with specific cut-offs for observables. The significance level was set at 0.05.

### Experimental procedures

The experiments and the reporting data are performed and published in accordance to the ARRIVE (Animal Research: Reporting of In Vivo Experiments) guidelines [28]. Fifteen newborn Yorkshire piglets (postnatal day 2 to 3; Willow Glenn Farm, Strasburg, PA) were studied. Animals were similar at baseline clinical characteristics. Animals were housed in standard cages and were exposed to 12-h light/12-h dark cycle. Access to standard newborn formula and water was provided during study ad libitum. The experimental protocol was approved by the Institutional Animal Care and Use Committee of Drexel University (IACUC protocol #200491). All methods were performed in accordance with the relevant guidelines and regulations. Appropriate use of drugs in standardized dosage was followed during experiments to reduce or eliminate pain, according to the NIH Guidelines for the Care and Use of Laboratory Animals [29]. Anesthesia was induced with 4% Isoflurane and maintained with 0.8–1% Isoflurane. The piglets were intubated within 10 min of induction of anesthesia and were placed on a pressure ventilator using a mixture of 75% nitrous oxide and 25% oxygen. Fluids and medication were administered using a peripheral intravenous catheter. Fentanyl (25 μg/kg) and pancuronium bromide (0.1 mg/kg) were administered through the intravenous route.

Following baseline ventilation with normal blood gasses for a period of 1 h, animals were randomly assigned to either a normoxic (Nx), hypoxic (Hx), or hypoxic treatment groups (Hx+PP2) by means of block randomization [30]. Hypoxia was induced by a gradual decrease of the FiO_2_ to 0.06 and maintenance at this level for 1 h.

Following 1 h of hypoxia, the cerebral cortex was harvested. Anesthesia and analgesia were maintained throughout hypoxia and brain harvesting through rapid midsagittal craniotomy. The parenchyma was flash-frozen in liquid nitrogen and stored at − 80 °C for further biochemical analysis. The piglets were maintained at 38–39 °C (normothermic). No animal died during the application of hypoxia. For the animals in treatment group, Src-inhibitor PP2 (1 mg/kg, IV) was administered a half-hour before Hx.

### Isolation of cerebral cortical neuronal nuclei

The isolation procedure for the cerebral cortical nuclei was based on the method of Giuffrida et al. [31]. One gram of brain tissue was homogenized in 15 volumes of a medium containing 0.32 M sucrose, 10 mM Tris–HCl, and 1 mM MgCl2 (pH 6.8). The resulting homogenate was then filtered through a nylon bolting mesh (size 110 lm) and subjected to centrifugation at 850 g for 10 min. To increases the yield of large neuronal nuclei, the homogenate was passed through a discontinuous gradient with a final sucrose concentration of 2.1 M, followed by another centrifugation step at 70,000 g for 1 h. The nuclear pellet was then collected and re-homogenized. Purity of neuronal nuclei was ensured by phase-contrast microscopy. Protein content was determined according to Lowry et al. [32].

### Isolation of the cytosolic fraction

One gram of cerebral cortical tissue was homogenized by a Dounce homogenizer (seven strokes with pestle clearance 0.15 mm and seven strokes with pestle clearance 0.07 mm) in 30 ml of fresh isolation medium (0.32 M sucrose, 1 mM EDTA, 20 mM Tris–HCl buffer, pH 7.1). The homogenate was then subjected to centrifugation at 1500 g, followed by a second centrifugation step at 15000 g for 10 min. The supernatant was finally centrifuged at 100,000 g for an hour to obtain the cytosolic fraction and the protein concentration was determined.

### Western blot analysis

Total protein extraction was performed in cell lysis buffer (0.5% NP-40, 0.5% SDS, 1.5 mM pH 7.4 Tris-HCL, 15 mM NaCl). Following electrophoresis (20 μg protein/lane) and polyvinylidene difluoride (PVDF) membrane transfer, primary antibody incubation was performed against Calcium Calmodulin Kinase dependent Kinase 2 [Sigma rabbit Anti-CAMKK2, Catalog# HPA017389, 1:500 dilution] overnight at 4 °C on a rocking platform. The membranes were treated with goat-anti-rabbit HRP-conjugated secondary antibodies (Invitrogen Thermo Fisher, Waltham, MA, USA). The target protein bands were determined using the reagents visualized provided in the ECL+ plus kit (GE Healthcare, Piscataway, NJ, USA). The immunoreactive band intensities were analyzed by imaging densitometry (GS 700 Imaging Densitometer, Bio-Rad) using ImageJ (National Institutes of Health, Bethesda, MD, USA). The protein expression is presented as optical density (OD) per mm^2^.

### Determination of ATP levels

Energy failure caused by hypoxia in cerebral cortex tissue was confirmed by determining the levels of ATP based on an assay previously described in detail [16, 33]. Briefly, frozen cortical tissue was powdered under liquid nitrogen in 6% w/v perchloric acid, thawed on ice, and then centrifuged at 2000 g for 15 min at 4 °C. The supernatant was neutralized using 2.23 M Potassium Carbonate/0.5 M triethanolamine/50 mM EDTA buffer (pH 7.3) and then subjected to centrifugation at 2000 g for 15 min at 4 °C. Three hundred microliters of the supernatant was added to 1 ml of buffer (50 mM triethanolamine, 5 mM MgCl2, 2 mM EDTA, 2 mM glucose, pH 7.6) and 20 μl NADP (10 mg/ml in 50 mM triethanolamine (TRA)-HCl buffer). Samples were incubated for 8 min with Glucose-6-phosphate dehydrogenase and read, following which Hexokinase was added and absorbance measured until steady state was reached. ATP concentration was calculated from the increase in absorbance at 340 nm and 20 μl each of ADP.

### Nuclear Ca^2+^ concentration

Neuronal nuclei (150 μg) were incubated in 300 μl medium composed of 50 mM Tris buffer (pH 7.4), 1 μM 45Ca^2+^, and 1 mM ATP. The Ca^2+^ influx assay was carried out at 37 °C for 2 min. Samples were then filtered on a glass fiber filter and washed three times in 20 mM Tris (pH 7.2), 100 mM potassium chloride buffer. A Rackbeta scintillation counter (Pharmacia, Gaithersburg, MD, USA) was used to measure radioactivity.

### Statistical analysis

Statistical analysis for experimental measurements was performed using Graphpad Prism v. 9. We used ordinary one-way ANOVA with Tukey post hoc multiple comparisons test. A *P*-value of less than 0.05 was considered statistically significant.

## Results

### CaMKK2 expression is reduced due to neonatal hypoxic brain injury

**Fig. 1:**
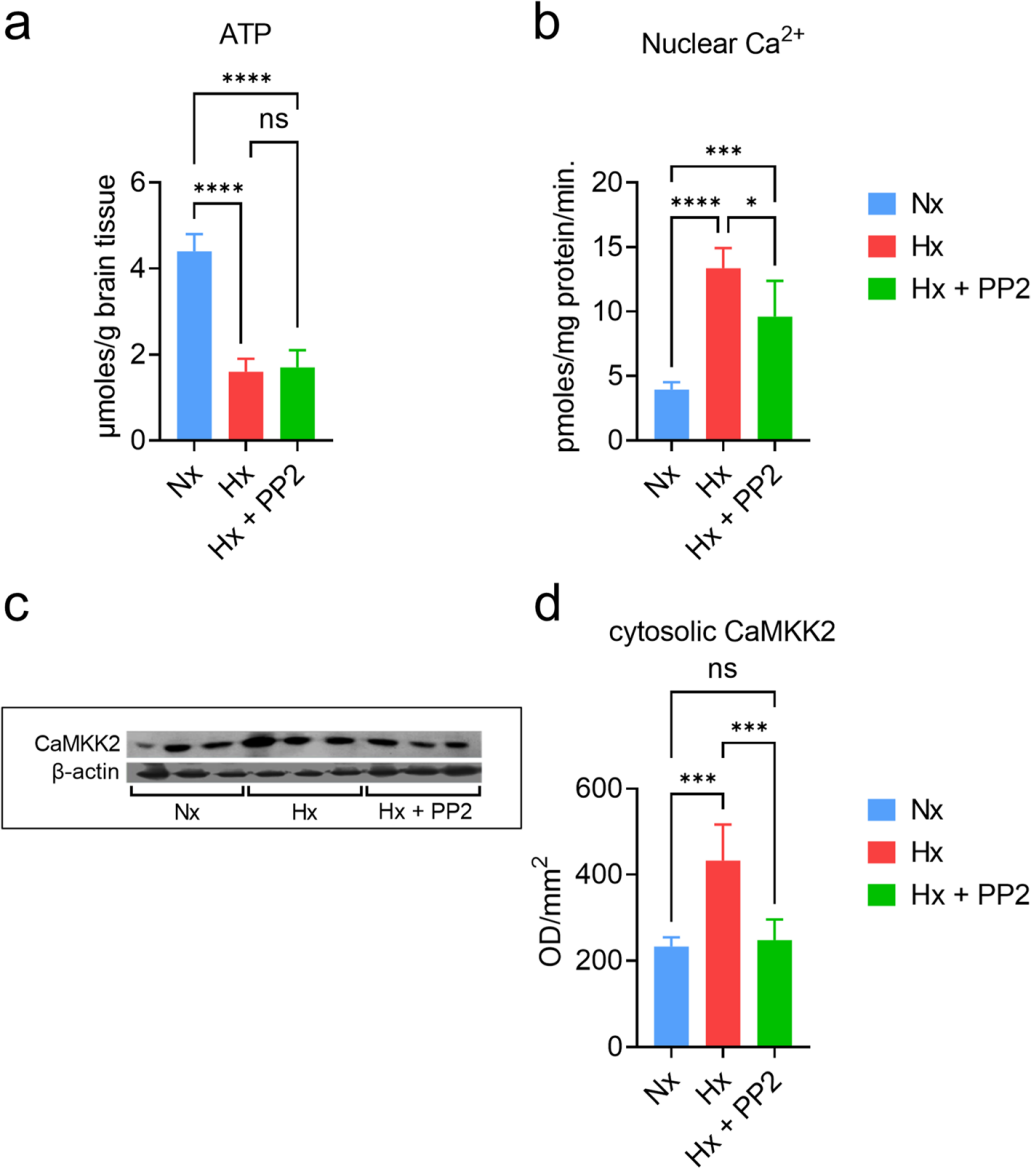
Experimental analysis of biochemical correlates and CaMKK2 expression in the piglet model of neonatal hypoxic brain injury. **a** ATP measurement in cerebral cortex tissue from piglets in the Nx (blue), Hx (red), and Hx+PP2 (green) groups; statistics: one-way ANOVA overall: *F* (2, 10) = 89.02 (*P* < 0.0001), Tukey multiple comparison test: Nx vs. Hx: *P* < 0.0001, Hx vs. Hx+PP2: *P* = 0.9253, Nx vs. Hx+PP2: *P* < 0.0001. **b** Nuclear Ca^2+^ influx in cerebral cortical tissue for all groups; statistics: one-way ANOVA overall: *F* (2, 14) = 32.94 (*P* < 0.0001), Tukey multiple comparison test: Nx vs. Hx: *P* < 0.0001, Hx vs. Hx+PP2: *P* = 0.0116, Nx vs. Hx+PP2: *P* = 0.0007. **c** Western blots of CaMKK2 for Nx, Hx, and Hx+PP2 groups, control: β-actin. **d** Quantification of CaMKK2 protein expression from western blots; statistics: one-way ANOVA overall: *F* (2, 15) = 19.68 (*P* < 0.0001), Tukey multiple comparison test: Nx vs. Hx: *P* = 0.0004, Hx vs. Hx+PP2: *P* = 0.0002, Nx vs. Hx+PP2: *P* = 0.9336

CaMKIV critically controls activity-dependent gene transcription in neurons [34]. We have previously shown that CaMKIV expression is significantly upregulated by neonatal hypoxic brain injury [35]. CaMKIV activity is strongly activated by CaMKK2 [34]. However, it remains unknown if CaMKK2 expression is also altered by to neonatal hypoxic brain injury. In order to identify the effect of hypoxia on the cerebral cortex, we first confirmed tissue hypoxia by measuring ATP (Fig. 1a). ATP was significantly reduced in both the Hx (*n* = 5) and Hx+PP2 group (*n* = 3) compared to Nx controls (*n* = 5) (one-way ANOVA—overall: *F* (2, 10) = 89.02, *P* < 0.0001; Tukey comparison Nx vs. Hx: *P* < 0.0001; Tukey comparison Nx vs. Hx+PP2: *P* < 0.0001). We also confirmed Ca^2+^ influx into neuronal nuclei (Fig. 1b), which increased in the Hx group (overall one-way ANOVA: *F* (2, 14) = 32.94; *P* < 0.0001; Tukey comparison Nx vs. Hx: *P* < 0.0001). PP2 treatment reduced hypoxic injury-induced Ca^2+^ influx (Tukey comparison Hx vs. Hx+PP2: *P* = 0.0116); however, Ca^2+^ levels in the Hx+PP2 group were still significantly higher than Nx (Tukey comparison Nx vs. Hx+PP2: *P* < 0.0001), indicative of a partial rescue. Western blot analysis (Fig. 1c) revealed that CaMKK2 expression significantly increased in the Hx group (Fig. 1d; overall One-way ANOVA: *F* (2, 15) = 19.68; *P* < 0.0001; Tukey comparison: Nx vs. Hx: *P* = 0.0004). However, in contrast to Ca^2+^ influx, CaMKK2 expression was restored to almost-normoxic levels (Tukey comparison: Hx vs. Hx+PP2: *P* = 0.0002; Nx vs. Hx+PP2: *P* = 0.9336). We used the changes in Ca^2+^ influx and alterations in CaMKK2 expression for both validation and predictive analysis for our computational model.

### Model validation and predictive analysis of pharmacological intervention with PP2

**Fig. 2:**
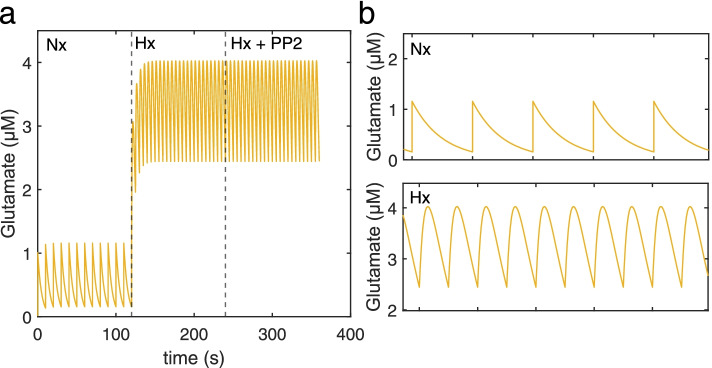
Model input. **a** Glutamate pulses during the three stages of the simulation—normoxia (Nx), hypoxia (Hx), and Hx+PP2. **b** Amplified view of Nx glutamate stimulation and **c** amplified view of Hx and Hx+PP2

**Fig. 3:**
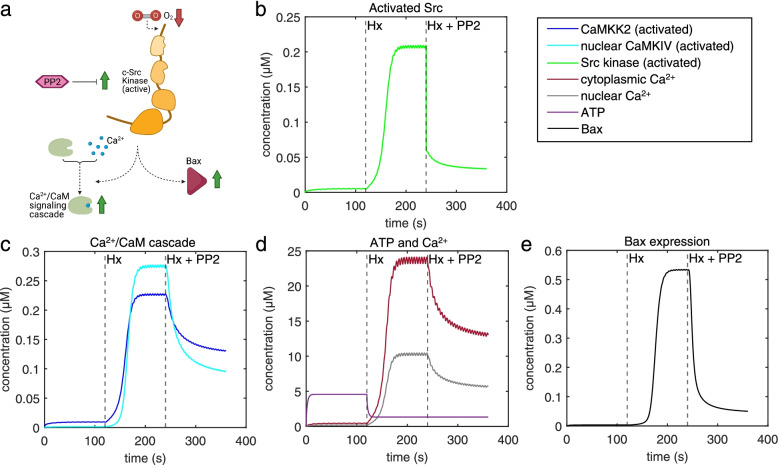
Ca^2+^/CaM-Src kinase model validation. **a** Cartoon schematic of Src kinase activation of the Ca^2+^/CaM signaling cascade and the pro-apoptotic protein—Bax—following hypoxic neonatal brain injury, as well as pharmacological inhibition of this pathway using PP2. **b** Simulation of our model yields activated Src dynamics **c** activated CaMKK2 (blue) and CaMKIV (cyan), **d** cytoplasmic (maroon) and nuclear Ca^2+^ (grey), ATP (purple), and **e** Bax (black) over simulation time course

We simulated model response to the input glutamate pulses under Nx, Hx, and Hx+PP2 conditions (Fig. 2). In accordance with previous experimental results, our model captured an increase in activated Src (Fig. 3a, b), nuclear CaMKIV (Fig. 3b), cytoplasmic and nuclear Ca^2+^ (Fig. 3c), and Bax expression (Fig. 3d) induced by Hx (dashed line at *t* = 120 s), and a decrease in concentration following Src-inhibition with PP2 (dashed line at *t* = 240 s). We validated our model with previously published data from Delivoria-Papadopoulos et al. [16] and from new data obtained from experiments we performed (Fig. 1; Table 1). Experimental trends for pharmacological intervention with PP2 matched trends between experimental data obtained from the piglet model and simulation results, with an effect accuracy of 40.22% for nuclear Ca^2+^, 69.01% for activated Ca^2+^/CaM, 89.56% for activated CaMKIV, and 47.56% for activated CREB (Table 1). While our model overestimated PP2 effect accuracies for nuclear Ca^2+^, activated CaMKIV, and activated CREB, it underestimated the PP2 effect for activated Ca^2+^/CaM. We then attempted to predict changes in alterations in CaMKK2 between Hx and Hx+PP2 conditions. Our analysis slightly underestimated the effect of PP2 intervention with an accuracy of 99.0%. Thus, based on the validation of our model, we proceeded to use it for further evaluation of perturbations to intracellular signaling induced by neonatal brain injury.

**Table 1:** Model validation and predictive analysis. Validation was performed on a dataset obtained from Delivoria-Papadopoulos et al., 2011 and our current work. Specifically, the effect of PP2 was used for validative analysis. The percentage change from Hx to Hx + PP2 conditions were calculated and compared between experimental and simulation data. Experimental trends were qualitatively assessed as match or mismatch. Effect accuracy was calculated based on comparison between PP2 effects as seen in experimental and simulation data. Depending on model prediction compared to experimental data, effects were labeled as either an ‘overstimate’ or ‘underestimate’

Molecule	Experimental data		Simulation data		Trend match/mismatch	Accuracy (Effect of PP2)
	Hx to Hx + PP2 (% change w.r.t Hx)		Hx to Hx + PP2 (% change w.r.t Hx)			
	change	trend	change	trend		
Validation analyses						
Nuclear Ca^2+^	-28.07	▼	-44.85	▼	Match	40.22%; overestimate
Activated Ca^2+^/CaM	-63.22	▼	-43.63	▼	Match	69.01%; underestimate
Activated CaMKIV	-59.10	▼	-65.27	▼	Match	89.56%; overestimate
Activated CREB	-36.84	▼	-91.20	▼	Match	47.56%; overestimate
Predictive analysis (test validation)						
Activated CaMKK(2)	-42.81	▼	-42.38	▼	Match	99.0%; underestimate

### Global sensitivity analysis

**Fig. 4:**
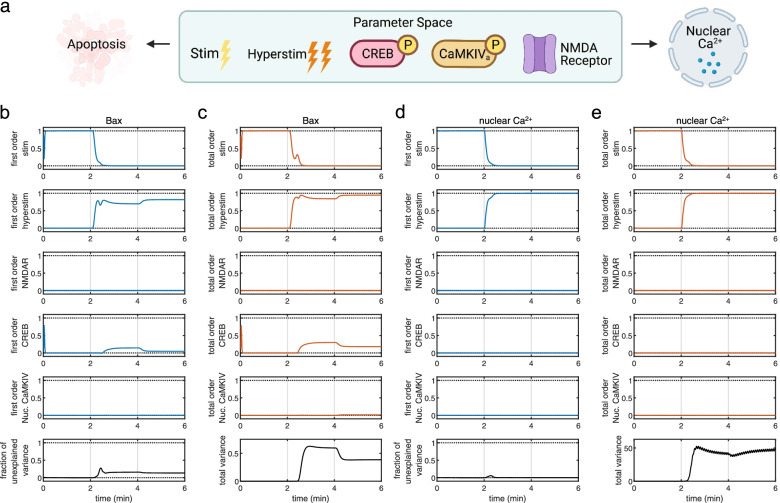
Global sensitivity analysis (GSA) of the Ca^2+^/CaM-Src model. **a** Input parameters include “stim” (normal glutamate pulse concentration), “hyperstim” (excitotoxic glutamate pulse concentration), NMDAR, CREB, and nuclear CaMKIV, with apoptosis (Bax) and Ca^2+^ influx as model readouts. **b** First-order Sobol indices for Bax expression. **c** Total-order Sobol indices for Bax expression. **d** First-order Sobol indices for nuclear Ca^2+^ influx. **e** Total-order Sobol indices for nuclear Ca^2+^ influx

We computed Sobol indices to analyze sensitivity of our model to changes in species concentration [27]. Based on literature and previously published results, we included “stim,” “hyperstim,” NMDAR, CREB, and nuclear CaMKIV, as input parameters. We varied parameters from 0.25X to 1.75X of starting concentrations. Observables included nuclear Ca^2+^ and Bax expression. Since we sought to measure both individual contributions of particular species parameters as well as interaction effects between parameters (Fig. 4a), we plotted first-order and total-order Sobol indices for model responses against simulation time course. We observed that, for Bax expression, stim (Nx glutamate pulse concentration; Fig. 4b–e, top panels), hyperstim (Hx glutamate pulse concentration, Fig. 4b–e, second-from-top panels), and CREB (Fig. 4b–e, fourth-from-top panels) displayed obvious first-order non-zero indices. Additionally, total-order indices for these parameters were higher than the corresponding first-order indices, indicating interaction between species parameters. Additionally, the fraction of unexplained variance (Fig. 4b–e, bottom-most panels) increased following the onset of Hx (grey vertical line at *t* = 2 min). Nuclear CaMKIV total-order indices during PP2 intervention (grey vertical line at *t* = 4 min) are slightly higher than first-order indices during the same time period, indicating the presence of interaction with other parameters. Surprisingly, for nuclear Ca^2+^ influx, only hyperstim showed slight interaction with other parameters (Fig. 4d–e). Specifically, within a few seconds following Hx onset, the fraction of unexplained variance indicated a slight transient increase. Thus, GSA for the chosen parameters indicates that our model is robust to changes in concentration of certain species including NMDAR and nuclear CaMKIV but is responsive to changes in normoxic and excitotoxic glutamate pulse concentration and CREB concentration. Importantly, these parameters interact such that there are differential effects on Bax expression and Ca^2+^ influx.

### Multi-parametric global sensitivity analysis

**Fig. 5:**
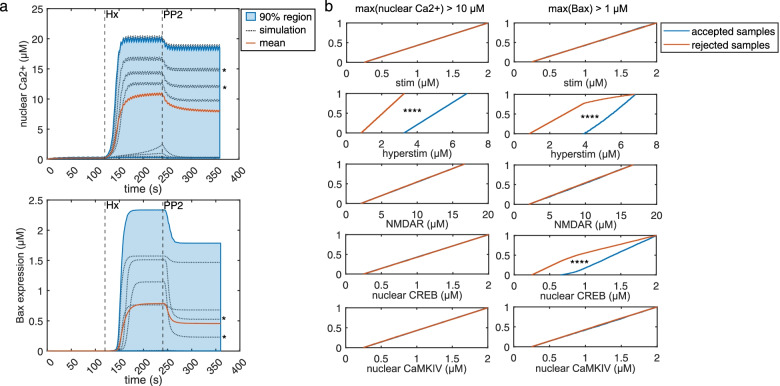
Multi-parametric global sensitivity analysis (MPGSA) of the Ca^2+^/CaM-Src model. **a** All simulation runs (dotted grey lines) over time course; mean model simulation response (red), and the 90% region (blue) for all simulation runs for nuclear Ca^2+^ influx (top) and Bax expression (bottom). **b** Cumulative distribution function plots for MPGSA with classifier max(nuclear Ca^2+^) > 10 μM (left) and max(Bax expression) > 1 μM (right), showing accepted (blue) and rejected (red) sample simulation values. Asterisks represents *P* < 0.0001 for Kolmogorov-Smirnov test to identify difference between accepted and rejected CDFs. [Statistics: (max(Bax) > 1 μM: CaMKIV: K-S: 0.0247; *P* = 0.9991; CREB: K-S: 0.4137; *P* < 0.0001; NMDAR: *P* = 0.9992, K-S: 0.0246; hyperstim: *P* < 0.0001; K-S: 0.7736; stim: *P* > 0.05; K-S: 0.0193; max(nuclear Ca^2+^ > 10 μM): CaMKIV: *P* > 0.05, K-S: 0.0139; CREB: *P* > 0.05, K-S = 0.0136; NMDAR: *P* > 0.05, K-S = 0.0153; hyperstim: *P* < 0.0001, K-S = 1; stim: *P* > 0.05, K-S = 0.0104]

In order to identify the particular conditions where input parameters of the GSA have differential effects on Ca^2+^ influx and Bax expression, we plotted all the simulations from the GSA (Fig. 5a). Two simulations revealed the dissociation between Bax expression and Ca^2+^ influx following PP2 administration (Fig. 5a, simulations [dotted lines] marked by asterisks in top and bottom panels). Since there was a discrete threshold above which simulations showed Ca^2+^-influx-Bax dissociation, we defined a classifier for multi-parametric global sensitivity analysis (MPGSA) based on a threshold for maximum values per simulation (maximum value > 10 μM for nuclear Ca^2+^ influx and maximum value > 1 μM for Bax expression). We reused the Sobol indices computed for the GSA for MPGSA and obtained empirical cumulative distribution functions for accepted or rejected simulations (Fig. 5b). Kolmogorov-Smirnov (K-S) tests between accepted and rejected samples indicated significant differences only for the hyperstim parameter for Ca^2+^ threshold. However, for Bax expression, we found significant differences for hyperstim as well as CREB [(max(Bax) > 1 μM: CaMKIV: K-S: 0.0247; *P* = 0.9991; CREB: K-S: 0.4137; *P* < 0.0001; NMDAR: *P* = 0.9992, K-S: 0.0246; hyperstim: *P* < 0.0001; K-S: 0.7736; stim: *P* > 0.05; K-S: 0.0193; max(nuclear Ca^2+^ > 10 μM): CaMKIV: *P* > 0.05, K-S: 0.0139; CREB: *P* > 0.05, K-S = 0.0136; NMDAR: *P* > 0.05, K-S = 0.0153; hyperstim: *P* < 0.0001, K-S = 1; stim: *P* > 0.05, K-S = 0.0104]. Thus, MPGSA further confirms that CREB and excitotoxic glutamate pulses significantly affect Bax expression while solely excitotoxic glutamate pulses can affect Ca^2+^ influx.

### Effect of NMDAR activation and Ca^2+^/CaM signaling kinetics on Ca^2+^ influx and Bax expression

**Fig. 6:**
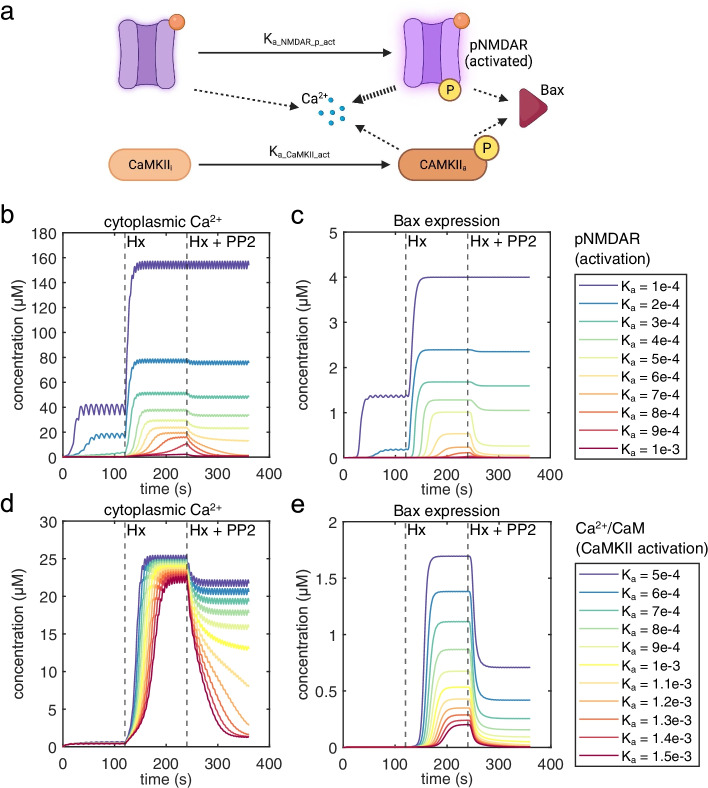
Parameter scanning to identify the relative dependence of signaling components. **a** Cartoon schematic showing Src-mediated NMDAR activation kinetics in comparison to CaMKII activation kinetics. **b** Dependence of NMDAR hyperstimulating activation kinetics on simulation outcomes for Ca^2+^ influx and **c** Bax expression. **d** Parameter scanning of CaMKII activation kinetics on simulation outcomes for Ca^2+^ influx and **e** Bax expression

Since activation of NMDAR and Ca^2+^/CaM signaling critically affects Ca^2+^ influx and activation of neuronal death pathways in neonatal hypoxic injury [36, 37], we performed parameter scans by altering the reaction kinetics of NMDAR activation (Fig. 6a). For NMDAR activation, our results showed that above *K*_a_ = 5e−4, Bax expression significantly drops during PP2 administration; however, a drop in Ca^2+^ influx is delayed, but reaches significant reduction above *K*_a_ = 7e−4 (Fig. 6b, c). This suggests that generally altering NMDAR activation only causes Ca^2+^-influx-Bax dissociation within a limited range. This is also indicative of a discontinuity in NMDAR signaling states that result in activation of cell death pathways. On the other hand, altering reaction kinetics of CaMKII activation results in a uniform relationship between Ca^2+^ influx and Bax expression during Hx (Fig. 6d, e). However, for *K*_a_ values ≤ 5e−4, we observe Ca^2+^-influx-Bax dissociation during PP2 administration.

### Effect of Src-NMDAR reaction kinetics and PP2-pre-treatment on Ca^2+^ influx and Bax expression

**Fig. 7:**
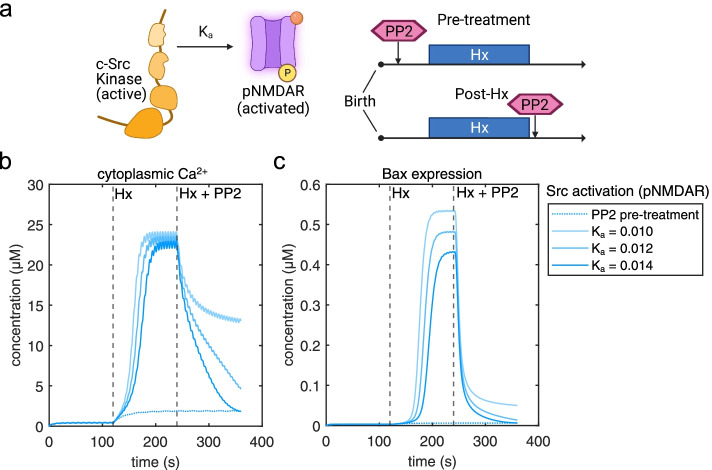
Effect of PP2 pre-treatment versus combination of PP2 administration following Hx and modulation of Src kinase activation of NMDARs (K_a_). **a** Cartoon schematic showing the effect of Src on NMDAR activation in relation to pharmacological treatment timeline. **b** Comparison of PP2-pre-treatment (dotted line) versus *K*_a_ = 0.01 (light tone blue), *K*_a_ = 0.012 (mid-tone blue), *K*_a_ = 0.014 (blue) on cytoplasmic Ca^2+^ influx and **c** Bax expression

Recent evidence strongly suggests that distinct modes of NMDAR signaling can dissociate channel Ca^2+^ influx and excitotoxic mechanisms in a Src-dependent manner [12, 13]. Additionally, it is clinically relevant to understand the temporal dynamics of Src inhibition and its interaction with NMDARs, which can lead to an attenuation of excitotoxic mechanisms. We compared PP2-pretreatment simulations with simulations where PP2 administration following Hx was combined with alteration of Src activation of NMDARs (Fig. 7a). While pre-treatment clearly prevented significant Ca^2+^ influx or increased Bax expression, increasing K_a_ of Src activation of NMDARs by just 20% (*K*_a_ = 0.012) resulted in a significant drop in cytoplasmic Ca^2+^ and returned Bax expression levels almost similar to pre-treatment (Fig. 7b, c). This indicates that while PP2 administration that follows Hx may still be beneficial, the effect may be dramatically improved in combination with small molecules that specifically alter Src-NMDAR reaction kinetics.

## Discussion

Drug design efforts are critically dependent on preclinical models of injury and disease. Often, these efforts are hampered by limitations of the animal models and their relevance to human anatomy and physiology. The piglet model of neonatal brain injury is a uniquely relevant system, since the developmental trajectory of the porcine brain shares many similarities to the human brain [38], including development of a gyrencephalic neocortex. Importantly, injury induced by hypoxic insult affects the piglet brain in a manner similar to that seen in human neonatal brain tissue [39]. Thus, it is not surprising that a significant percentage of studies on neonatal brain injury employ the piglet model [40]. However, while few computational studies have used the piglet model to analyze the effect of neonatal brain injury on cerebral blood flow and metabolism [41–43], computational approaches to study intracellular signaling in normal and injured states has not been performed in this model. Since this approach has generated novel insights into neuronal function based on data from rodent models [44–46], our study represents a significant step in building computational models of intracellular signaling with greater translational relevance to human disease and injury conditions.

We have built a computational model of the Ca^2+^/CaM-Src-kinase signaling pathway in the context of neonatal brain injury induced by Hx. The present computational analysis is primarily based on experimental data obtained from a translational large animal model of neonatal brain injury highlighting the clinical relevance of our model. Simulations from our model capture the molecular changes observed in Hx, which we validated from previously published experimental work (Table 1, Fig. 3). The present model also enables predictive analyses in determining the effect of pharmacological modulation of Src on different signaling components (Table 1). GSA (Fig. 4) and MPGSA (Fig. 5) of our model determined that differential interaction between input parameters results in dissociation of Ca^2+^ influx and Bax expression. Parameter scanning of reaction kinetics indicated that activation of NMDARs (Fig. 6), and specifically Src activation kinetics of NMDARs (Fig. 7) modulate dissociable signaling states between Ca^2+^ influx and Bax expression. Finally, simulations of our model strengthened the hypothesis that PP2 inhibition of Src kinase ameliorates the effect of neonatal brain injury and that modulating Src-NMDAR reaction kinetics is a potential therapeutic strategy in attenuating the expression of apoptotic pathway proteins (Fig. 7). Thus, our computational approach of modeling excitotoxic mechanisms in cortical neurons of the piglet model of neonatal brain injury provides a translational platform for designing and screening drugs targeting interactions between glutamate receptors and their functional modulators, such as Src kinase.

The dominant paradigm to explain excitotoxic neuronal damage involves an abnormal influx of Ca^2+^ via NMDARs and AMPA/kainate receptors. The large Ca^2+^ influx then activates multiple signaling cascades leading to dysfunctional mitochondrial metabolism and the formation of reactive oxygen species (ROS) [47]. Since hypoxic conditions limit ATP production, this further weakens neurons’ ability to counter an excitotoxic event. In our model, a reduction in ATP production has been programmed as being triggered by a reduction in extracellular O_2_, in line with our previous work where ATP levels are used as a readout of tissue hypoxia [16]. Activation of ROS and other cell death and toxicity-related pathways leads to the formation of proapoptotic proteins like Bax and Bcl-2, which eventually leads to mitochondrial-dependent damage and nuclear DNA fragmentation. In our model, we have focused on the proapoptotic protein Bax as a final readout of excitotoxic damage. However, Ca^2+^-mediated excitotoxic mechanisms can also lead to neuronal necrosis, which is a considerably faster form of cell death. It is likely that severe hypoxic conditions can lead to both necrosis and apoptosis. Evidence also suggests that in models of neonatal brain injury, hypoxia may induce both these forms of cell death on a continuum, rather than completely discrete events without any overlap [2]. Although apoptosis is a slower form of neuronal death, the molecular processes that eventually lead to death are well underway within minutes of hypoxic/ischemic insults [48, 49], and Bax translocation to mitochondria also occurs on a relatively rapid timescale [50]. Thus, although the timescale of Bax expression in our model is relatively fast, there is precedent for a comparable timescale from previously published work.

Excitotoxic mechanisms have been studied in the context of hypoxic brain injury for a considerable period of time. However, few if any effective pharmacological agents that attenuate neuronal damage induced by excessive glutamatergic excitation have been identified. Our previous work strongly indicates that the Src kinase inhibitor PP2 is an effective intervention to reduce Hx-induced excitotoxic neuronal damage in the piglet neocortex [19, 20]. Our previous work also suggests that this neuronal damage is initially triggered by the activation of the Ca^2+^/CaM pathway that cascades through a series of intermediate steps, including the activation of CaMKII, CaMKK2, nuclear CaMKIV, and finally CREB. However, the GSA and the MPGSA of our computational model suggest that, although excitotoxic hyperstimulation shows significant interaction with key signaling members of this cascade, the only other critical component that significantly interacts with excitotoxic hyperstimulation is CREB. This indicates that CaMKIV, while important, may not be a limiting component of excitotoxic mechanisms that signal via Src kinase. Another important insight from the GSA and the MPGSA points to the existence of different and discrete signaling states, where Ca^2+^ influx and Bax expression can be dissociated during Src inhibition. This is in line with emergent evidence on metabotropic-like signaling of NMDARs triggered by excitotoxic stimuli, wherein cell death can be dissociated from Ca^2+^ influx per se [12, 13]. Interestingly, since Src kinase interaction with NMDARs during excitotoxic damage is mediated by Panx1 channels [13], it would be useful in future studies to also include Panx1 as part of the intracellular signaling mechanism in our model.

An important contribution of our work is the establishment of a computational platform to analyze pharmacokinetics and pharmacodynamics of drugs targeted to reducing neuronal damage induced by hypoxic excitotoxic mechanisms. In our study, the Src inhibitor PP2 served to define the role of Src kinase in the pathway. However, a key difference between our previous studies and our current model simulations is that in previous studies comparison between groups included PP2 pre-treated piglets exposed to Hx, whereas in our model, PP2 was administered 2 min after Hx. In spite of this difference, the model gave us an opportunity to compare pre-treatment with PP2 against post-Hx administration. Although altering CaMKII activation results in modest reduction in Ca^2+^ influx but does not significantly alter a drop in Bax expression, modulating Src activation of NMDARs in addition to PP2 administration caused a significant reduction in both Ca^2+^ influx as well as Bax expression (Fig. 6). Thus, our model indicates that designing or screening small molecules that effectively mimic the activation of NMDARs by Src kinase could be a potential therapeutic strategy in mitigating excitotoxic neuronal damage.

### Study limitations

This study, although addressing the important problem of excitotoxic mechanisms in cortical neurons of the piglet model of neonatal brain injury, has some limitations. For the purpose of the computational analysis, we designed our model to specifically address excitotoxic Ca^2+^ influx via NMDARs. Although our model does include other sources of Ca^2+^ influx into the cytoplasm and nucleus, including CP-AMPARs, VGCCs, and the ER, we have not included detailed regulatory pathways and mechanisms for these sources, which minimized their contribution to the overall dynamics of Ca^2+^ influx. Although sources such as VGCCs may play a minor role in neuronal injury when compared to NMDARs [51], a more detailed model including other Ca^2+^ sources may enable testing competing hypotheses regarding the role of Ca^2+^ in excitotoxicity, such as the “Ca^2+^-loading” hypothesis [52] and the “source specificity” hypothesis [53].

The Ca^2+^/CaM signaling cascade involves multiple kinases including some which have been included in our model (CaMKII, CaMKIV, and CaMKK2). The interaction between these kinases affects multiple signaling pathways. CaMKK2 in particular serves as a hub integrating and decoding signals across diverse pathways [54–56], while also being subject to regulatory mechanisms including modulation and autoinhibition [57, 58]. However, these mechanisms have not been completely characterized. Thus, in our model, we have included a simplified version of the Ca^2+^/CaM signaling cascade. Future computational studies could shed light on these kinome-related mechanisms and how they affect the Src kinase pathway in the context of neonatal brain injury.

In the present study, we have not included a detailed model of mitochondrial metabolism and how changes in extracellular O_2_ during Hx may affect these dynamics and feedback onto the Src kinase pathway. In addition, we have not included a mitochondrial Ca^2+^ buffering mechanism, which is likely altered in conditions that lead to neuronal death [59–61], although these mechanisms are downstream of our final readout of Bax expression.

Importantly, our model input is a physiologically very slow rate of glutamate release and captures slower components of excitotoxic release. It would be helpful to use faster glutamate release dynamics in future studies and adjust other temporal kinetics of the model accordingly.

Finally, we did not include a comprehensive sub-model of apoptosis in our approach. Future studies should also include the expression of the pro-apoptotic protein Bad as well as anti-apoptotic proteins, such as Bcl-2 and Bcl-xl, for which experimental data is available in the piglet model of neonatal brain injury [17].

## Conclusions

We have used experimental and computational approaches to determine the significance of the Src-Ca^2+^/CaM pathway in neuronal excitotoxicity in a large animal model of neonatal hypoxic brain injury. Our computational model was validated with experimental measurements of critical intracellular signaling components and captures key molecular trends in this pathway. Our computational model indicates that pharmacological inhibition of Src kinase leads to signaling states wherein Ca^2+^ influx and the onset of Bax expression are dissociable. Finally, our computational model predicts that small molecules that specifically modulate the interaction between the NMDA receptor and Src can significantly contribute to a reduction in Bax expression. Our study combines experimental and computational methods to provide a translational platform to investigate key signaling cascades triggered by neonatal hypoxic brain injury in neurons.

## Supplementary Information

**Additional file 1: Supplementary Figure 1.** Representative visualization of Ca^2+^/CaM-Src kinase intracellular signaling computational model. Cartoon depiction of the interactions captured in our SimBiology model of excitotoxic glutamate pulses and subsequent activation of NMDARs and Ca^2+^/CaM signaling through Src kinase and the eventual transcription of the pro-apoptotic protein Bax. Figure created with BioRender.

**Additional file 2.**

## Data Availability

The SimBiology Project file (“Kratimenos_et_al_CaM-Src_model_FINAL.sbproj”) which contains the model has been uploaded to the public repository Zenodo.org and can be accessed here: 10.5281/zenodo.5976575. Manuscript data for figs. 1, and 3-7 can be accessed here: 10.5281/zenodo.6349288.
