# Supplementary figures and images for: Computational analysis of cortical neuronal excitotoxicity in a large animal model of neonatal brain injury
Source: J Neurodev Disord. 2022 Mar 29;14:26. doi: 10.1186/s11689-022-09431-3 (PMC8966144; doi:10.1186/s11689-022-09431-3)

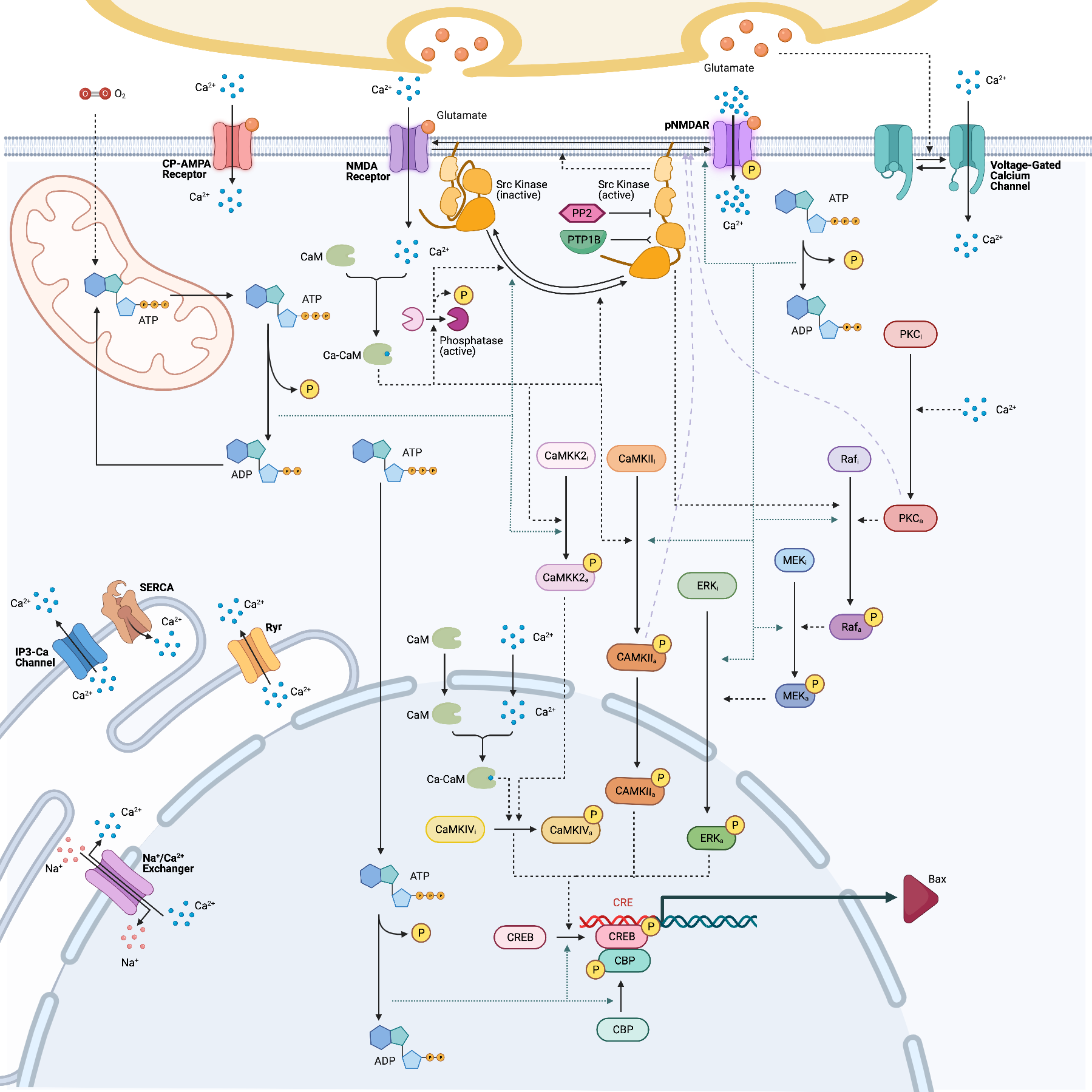

Supplement: Supplementary file 1 — Additional file 1: Supplementary Figure 1. Representative visualization of Ca2+/CaM-Src kinase intracellular signaling computational model. Cartoon depiction of the interactions captured in our SimBiology model of excitotoxic glutamate pulses and subsequent activation of NMDARs and Ca2+/CaM signaling through Src kinase and the eventual transcription of the pro-apoptotic protein Bax. Figure created with BioRender. [file 11689_2022_9431_MOESM1_ESM.png]
